# Multimodal MRI in the course of cardiac wound healing after myocardial infarction

**DOI:** 10.1186/1532-429X-14-S1-P33

**Published:** 2012-02-01

**Authors:** Florian Bönner, Christoph Jacoby, Ulrich Flögel, Sebastian Temme, Nadine Borg, Malte Kelm, Jürgen Schrader

**Affiliations:** 1 Department of Cardiology, Pneumology and Angiology, University Hospital of Heinrich Heine University, Düsseldorf, Germany; 2 Department of Molecular Cardiology, Heinrich Heine University, Düsseldorf, Germany

## Background

Myocardial tissue undergoes various adaptive processes in the course of healing after myocardial infarction including infiltration of immunocompetent cells. Assessment of myocardial infarction and subsequent healing with contrast-enhanced and T2-weighted MRI holds the advantage of non-invasive localisation and characterisation of myocardial lesions, determination of their functional relevance and monitoring oedema formation and resorbtion in myocardial tissue. A quantitative method to determine immune cell migration into myocardial tissue as consequence of myocardial infarction is provided by 19F MRI after intravenous application of emulsified perfluorocarbons (PFCs).

## Methods

For induction of myocardial infarction C57Bl/6 mice underwent 50 min of coronary ligation with subsequent reperfusion. MRI measurements were carried out with a DRX 9.4 T NMR spectrometer (Bruker, Rheinstetten, Germany). Cine imaging for cardiac function analysis was performed using a respiration- and ECG-gated gradient echo sequence. For contrast-enhanced measurements a T1 weighted gradient echo sequence was used to detect myocardial necrosis after contrast agent application (Gadolinium DTPA). T2 maps were calculated from gated multi spin echo images. All measurements were performed at baseline, 1, 7, 14 and 28 days after I/R. Infiltration of immune cells was measured by 19F MRI (multislice RARE sequence, factor 64) at days 3 and 7 after I/R and intravenous injection of 500µl PFCs 24 hrs prior to MRI.

## Results

**Figure 1:**
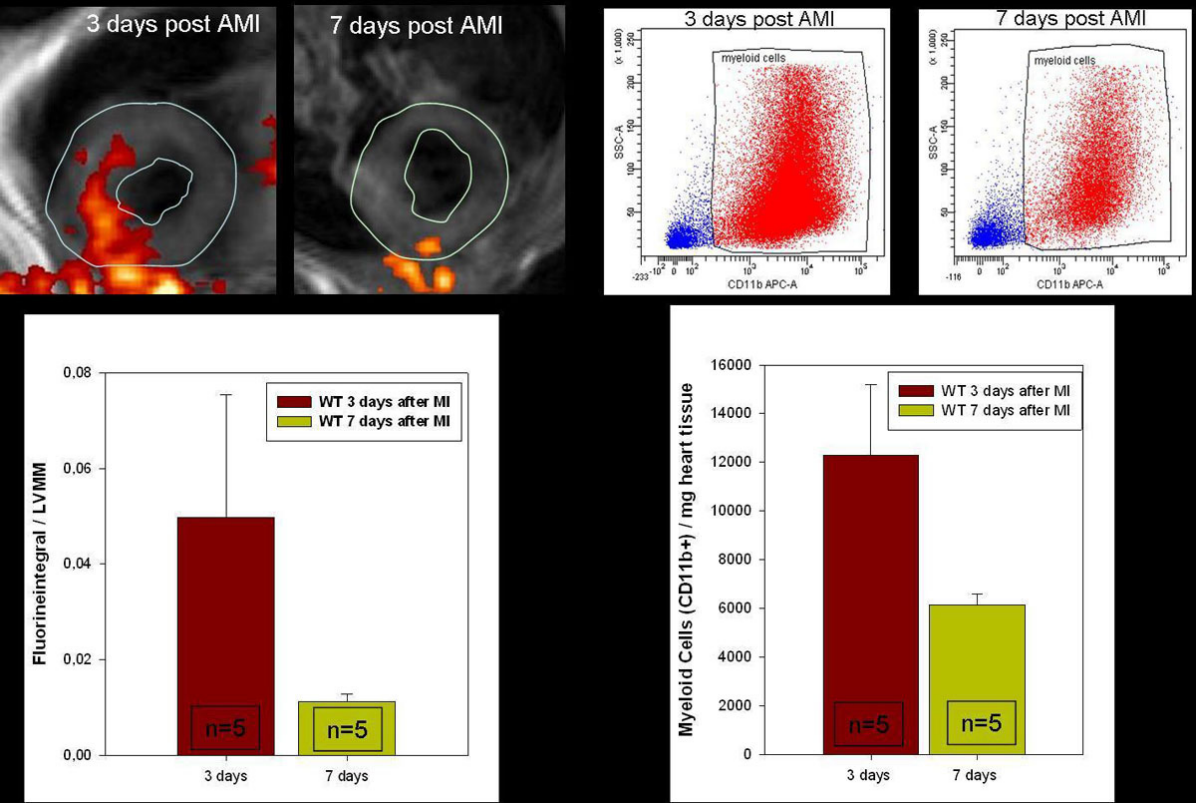


Necrotic areas correspond well with regions of highly elevated T2. Functional impairment as measured by regional wall movement analysis showed a close relation to areas with increased T2 (r=0.90). Within the observation period of 28 days, T2 values of myocardial tissue returned to baseline levels. This was found to be accompanied by improvement of ventricular function. Most interestingly, this observation was associated with a reduction in total fluorine intensity within the left ventricle at day 7 compared to day 3 after I/R. This observation was confirmed by quantitative myocardial immune cell analysis with flow cytometry (Figure 1).

## Conclusions

In summary, multimodal MRI after I/R provides complementary information and therefore holds promise for serial analysis of myocardial tissue characteristics. Since all measurements can be performed with one setup, no repositioning of the animals is required enabling an exact overlay of identical myocardial regions. The combination of cine imaging, late enhancement and T2-mapping techniques with 19F MRI enables a comprehensive analysis of functional, morphological and inflammatory properties of the healing myocardium after infarction in vivo.

## Funding

None.

